# Traffic Games: Modeling Freeway Traffic with Game Theory

**DOI:** 10.1371/journal.pone.0165381

**Published:** 2016-11-17

**Authors:** Luis E. Cortés-Berrueco, Carlos Gershenson, Christopher R. Stephens

**Affiliations:** 1 Posgrado en Ciencia e Ingeniería de la Computación, Universidad Nacional Autónoma de México, Ciudad de México, Distrito Federal, México; 2 Instituto de Investigaciones en Matemáticas Aplicadas y en Sistemas, Universidad Nacional Autónoma de México, Ciudad de México, Distrito Federal, Mexico; 3 SENSEable City Lab, Massachusetts Institute of Technology, Cambridge, Massachusetts, United States of America; 4 MoBS Lab, Northeastern University, Evanstone, Illinois, United States of America; 5 ITMO University, St. Petersburg, Russian Federation; 6 Instituto de Ciencias Nucleares, Universidad Nacional Autónoma de México, Ciudad de México, Distrito Federal, México; 7 Centro de Ciencias de la Complejidad, Universidad Nacional Autónoma de México, Ciudad de México, Distrito Federal, México; University of California Irvine, UNITED STATES

## Abstract

We apply game theory to a vehicular traffic model to study the effect of driver strategies on traffic flow. The resulting model inherits the realistic dynamics achieved by a two-lane traffic model and aims to incorporate phenomena caused by driver-driver interactions. To achieve this goal, a game-theoretic description of driver interaction was developed. This game-theoretic formalization allows one to model different lane-changing behaviors and to keep track of mobility performance. We simulate the evolution of cooperation, traffic flow, and mobility performance for different modeled behaviors. The analysis of these results indicates a mobility optimization process achieved by drivers’ interactions.

## Introduction

The dynamics of vehicular traffic, independently of its type (urban, freeway, one car system, mono-laned, multi-laned), is a paradigmatic example of a complex adaptive system [1–5]. As such, it is difficult to model. The principal paradigm that has been used to model vehicular traffic is as a physical flow of objects–vehicles–that interact in different ways. It is this paradigm that is at the heart of most academic studies and, indeed, the different techniques and technologies for the design of Traffic Control Systems (TCS)[6–9]. These physics-based models focus their attention on the interactions vehicle-TCS, or physical interactions between vehicles, assuming semi-idealized driver behavior, disregarding human unpredictability and behaviors that may help to self-organize other drivers [4]. In short: they do not account for the fact that vehicles are controlled by drivers, who make decisions and adopt different strategies in order to achieve their goals. Of course, representing the appropriate degree of heterogeneity among drivers is a difficult modeling problem. Too little will lead to limited results by oversimplifying the drivers’ behavior. The performance of Traffic Control Systems (TCS) based on studies with limited results, due to the oversimplification of drivers, may be affected by perturbations caused by real drivers’ behavior.

Studying vehicle-vehicle interactions implicitly, *i*.*e*., induced by the decisions they make, is not trivial, offering an open field for answering questions such as: How is the efficiency of already proposed TCS solutions affected by perturbations caused by drivers’ behaviors? What is the nature of the interactions between drivers? Are we able to model a more realistic behavior?

There are some traffic models that tackle some “social” (driver-driver interactions) aspects. The Wastavino *et al*. crossroad model [10] analyzes, among other important issues, the different effects that drivers may cause by cautiously or aggressively crossing an intersection. The MOBIL lane-changing model [11] includes a politeness factor that dictates how much drivers will care about the conditions of the other drivers while lane changing thus influencing the rate of lane changes. There are some studies, like the one performed by Nakata *et al*., about a Prisoner’s Dilemma-type game structure within the traffic flow [12]. The results of these contributions highlight the importance of including a more realistic representation of drivers’ behavior in traffic models.

An important step in including driver-driver interactions and the associated heterogeneity is choosing an appropriate framework. Here we will develop and use an agent-based/game theory approach. Decades ago, game theory was created as a tool to help us to understand decision making processes [13][14]. It has been used to study very particular decision making processes, such as auctioning or the formation of oligopolies, voting systems [15], fair division [16], war strategy [17], animal communication[18], animal mobbing behavior [19], *etc*. It has also been used as a framework in which to understand more generic phenomena associated with decision making, such as the evolution of fairness [20], evolution of cooperation[21], evolutionary dynamics of social dilemmas [22] and governance of risky commons [23]. There exist many game-theoretic studies that focus their attention on the decision-making processes involved in urban traffic [24,25], from macro policies analysis (between authorities, between drivers and authorities, and between drivers) to micro behavior descriptions (between drivers and authorities, and between drivers).

Driving can clearly be described as a decision-making process. Once a vehicle moves, the driver takes multiple decisions, such as when to change lane, when to brake, and when to wait or cross a yield signaled intersection [10]. There are studies focused on describing many of these decisions using the game theory paradigm and applying these descriptions to autonomous vehicles [26] and vehicle to vehicle (V2V) communication [27] scenarios. These types of models generally assume agents with perfect rationality, capable of determining which action will lead to an optimal payoff for her and the other drivers with whom she is interacting. For autonomous vehicle scenarios, defining which actions will lead to optimal benefits for every participant is an important task. However, scenarios involving human drivers may need another approach. It has been pointed out [25] that the behavior of human drivers may take into account other factors besides rationality and also drivers may not have all the information needed to calculate an optimal payoff while driving.

The use of agents with complete rationality may have another drawback [25]: In order to obtain optimal solutions, these models use powerful but computationally expensive algorithms. These kind of models also suffer the, already mentioned, difficult modeling problem of representing an appropriate degree of heterogeneity among drivers. Too much will lead to overly complicated, difficult to interpret models, *e*.*g*. sometimes the models must be simplified to make them computationally practical [27] and offer results. This characteristic prevents this type of models from being used as the basis for real time TCS.

Our goal is to analyze the impact of different drivers’ behaviors on traffic performance. On the one hand, we have models with semi-idealized drivers’ behavior with possibly limited results. On the other hand, we have models with completely rational drivers that may not represent real drivers and that use computationally expensive algorithms. To achieve our goal, we need models that balance the two types of model characteristics already discussed. In this paper, we present a model that falls into this description. It consists of agents with bounded rationality that are able to choose between two possible behaviors (cooperative or defective) while driving on a freeway. Such a model could help us to identify which drivers could be doing something “right” and those which could be doing something “wrong”. Thus, we can understand and try to change what is wrong and analyze if it is possible to translate what are they doing right to other scenarios, which could include autonomous vehicles.

In the next section (Development of the new model) we will discuss the conceptualization of how to integrate the evolution of cooperative behavior in a constant population of agents, as described in [28], to a traffic simulator that includes lane-changing maneuvers [29]. These agents are capable of self-organizing themselves to demonstrate the evolution of cooperation, as achieved by the life-cycle based agents of [21]. The result is a traffic simulator that includes lane-changing behavior that is based on the evolution of cooperation that, in turn, lets us study the performance of different modeled behaviors with additional information.

In the Method section we specify all the modifications made to state of the art traffic models [29] to obtain our new model. We also specify all the parameter values used to obtain the results shown in this paper. In the Results section we compare the performance of the original model [29] against the performance associated with the different behaviors introduced in this paper. In the last section, results are discussed in terms of traffic performance and in terms of the evolution of cooperation.

## Development of the New Model

Many correct driving behaviors (using designated lanes, crossing signaled intersections only when the green light is lit, using directional lights at lane changes, facilitating other’s lane changes, *etc*.) may be labeled as cooperative interactions in which one driver pays a cost (time, speed, space) and another driver receives a benefit [21]. Drivers avoid crashing most of the time because of these interactions.

This characterization of driving could help us to create a game-theoretic framework in which to model and study different phenomena while taking into account human factors. Also, the creation of this framework could open new possibilities for the application of game-theoretic models to spatial problems.

In order to create the framework in which we will study the impact of driver decision making on the dynamics of a given traffic scenario, we need to implement a traffic model. As we pretend to model and study drivers’ interactions, a microscopic traffic model, such as the NaSch model [1], is appropriate. It is known that this cellular automaton model replicates the fundamental diagram for a single lane freeway by defining key vehicle drivers’ behaviors, *i*.*e*., acceleration, braking and random braking. Although this is a powerful model, we need a more realistic representation of the drivers if we want to define a cooperative characterization of the driving process for example.

Considerable effort has been invested in the development of more realistic cellular automata models [30]. These models include limited deceleration capacity [31], limited capabilities of acceleration and deceleration, realistic safe distances between following and leading vehicles [32] (hereafter referred as the LAI model) and lane changing interactions between heterogeneous vehicles [29] (hereafter referred as the GLAI model).

**Fig 1:**
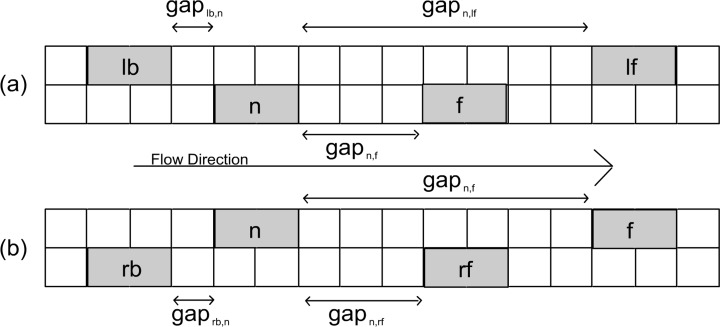
Key variables for vehicle *n* during a lane change. (a) On a left-lane-change a vehicle *n* must consider the vehicle in front of it *f*, the vehicle in front and at left *lf* and the vehicle behind and at left *lb*. (b) On a right-lane-change a vehicle *n* must consider the vehicle in front of it *f*, the vehicle in front and at right *rf* and the vehicle behind and at right *rb*.

The GLAI model [29] brings together all of these realistic features with a simple list of key variables (Fig 1).

Using information from other vehicles (velocity and position), each vehicle calculates threshold distances that dictate in which circumstances a vehicle may accelerate, has to keep its speed, has to brake or must use the emergency brakes [32].

Both key variables and threshold distances are necessary when a vehicle is going to change lane [29]. A lane change will take place when a vehicle determines that the conditions are beneficial (incentive conditions). For example: (1) while driving on the right lane, if the conditions of the lane dictate that the vehicle has to keep its speed but the conditions of the left lane are favorable for accelerating; (2) While driving on the right lane, if the conditions of the lane dictate that the vehicle has to brake, or must use emergency braking, but the conditions of the left lane are favorable for at least maintaining its speed; (3) While driving in the left lane, whenever the conditions of the right lane make it possible.

Once a vehicle determines that conditions are favorable, it changes lane only if the safety condition is met, *i*.*e*. the gap between the vehicle changing lane and the vehicle in the other lane and behind must be larger than the distance that the vehicle in the other lane and behind will travel using emergency braking.

As it may be observed, the GLAI model abstracts a two-lane freeway with asymmetric lane changes (left lane change incentive conditions are different from the right lane change incentive conditions) without collisions using the cellular automata paradigm. Taking advantage of the similarity between cellular automata models and agent models we merge the GLAI model with a game-theoretic agent model [28] in which the agents adapt themselves to their social environment. Another important reason to use the GLAI model, besides the fact that it successfully models safe distances between vehicles based on their relative velocity and realistic acceleration/deceleration rates, is that the GLAI model has incentive and safety conditions for lane changes that include variables that facilitate the addition of new elements which can be used to include decision-making elements in the sections of the simulator that are related to lane change maneuvers. The agents may choose to be more cooperative or to be more selfish depending on the payoff obtained, while iteratively playing games proposed for the study of the evolution of cooperation [21]. We choose to label the actions of the drivers as cooperative (cautious behavior or behavior with strict adherence to regulations) or defective (risky behavior or behavior with loose adherence to regulations) to be consistent with the rules whose behavior we are going to analyze. Theses labels may be changed to other ones though we believe this labeling is useful since many regulations are based on the fair use of traffic infrastructure.

**Fig 2:**
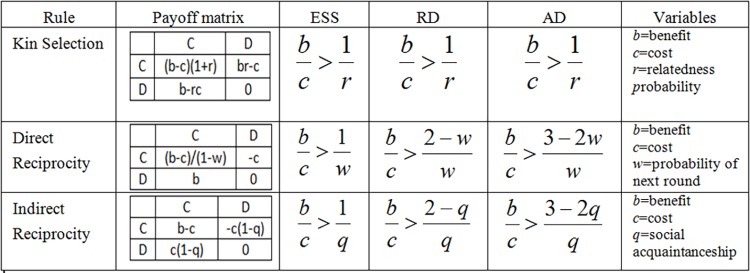
Three mechanisms for the evolution of cooperation described as a payoff matrix specifying the conditions in which cooperation is an evolutionary stable strategy (ESS), risk dominant (RD) or advantageous (AD) in comparison to defection.

In these games (Fig 2), as explained in [21], the players who choose to cooperate are going to pay a cost (*c*) that the other player is going to receive as a benefit (*b*). In our model, the values for *c* and *b* are given by the velocity increase or decrease that is generated as a result of a vehicle lane change. Each rule focuses on a particular feature and explains how this feature favors the evolution of cooperation.

**Kin Selection:** With this rule, the feature under analysis is “genetic” relatedness (*r*). While the probability of being genetically related to another player increases, so will the cooperative tendency of the whole population. The idea behind this mechanism is that cooperation is more likely between “relatives” rather than “non-relatives”. In [21] “relatives” refers to a pair of agents that share a gene. Mapping this scenario to freeway traffic situations, “relatives” may refer to social acquaintances (coworkers, neighbors, friends). The concept of “relatives” may differ between cultures; highly cooperative cultures may recognize any other fellow citizen as a “relative” given the premise that each citizen has the same right to make use of the freeway. Following this thinking, different cultures may have different relatedness probability values (*r*).

**Direct Reciprocity:** For this mechanism, the memory of the agents is taken into account. The players born as cooperators are going to implement a tit-for-tat behavior, cooperating on the first move. The players born as defectors always defect. With these conditions, the probability of playing again with a particular agent (*w*) becomes a key feature for the evolution of cooperation. Mapping this situation to a freeway traffic context seems to be pointless as the probability of the same two drivers playing (interacting in a lane change) is very low, except during high density situations (jam phase). However, it has been demonstrated that during high density situations few lane changes occurs [29], we therefore decide not to map this scenario in our model.

**Indirect reciprocity**: In this case the feature that promotes the evolution of cooperation is the social acquaintanceship (q) of the players’ acts. Players born as defectors will always defect. Players born as cooperators are going to try to identify their game partner as a cooperator or as a defector. The probability of the cooperators successfully identifying their game partners is equal to the fraction of the population able to perceive their actions (social acquaintanceship). If a cooperator successfully identifies the other as a defector then the player will defect, otherwise cooperators will always cooperate. On a freeway, drivers can categorize the behavior of other drivers as cooperative or defective by observing the interactions of such drivers with others. Although this identification is useful for a short time, it may be that under other conditions (traffic in small communities) it may be useful for longer periods. Also this rule could be easily adapted to represent V2V communication scenarios where the communications could be modeled by the social acquaintanceship. For this reason we consider it interesting and we include it in our studies.

Note that in the three original rules [21], players are born as cooperators or defectors. They cannot change their affiliation between games. They are alive for a certain amount of time and then they die, leaving offspring that receive their affiliation as inheritance and with a given probability that this affiliation may mutate. This formulation for the evolution of cooperation does not seem that suitable for freeway traffic situations however, and a more straightforward approach may be more useful.

In [28] we have the same rules (Kin Selection, Direct Reciprocity and Indirect Reciprocity), applied over a constant number of players who start with a certain degree of attachment for cooperation or defection and who may change this degree according to their convenience. Players also start with a certain amount of an abstract resource that may be lost or gained in each game. Each agent tries to maximize this resource by applying simple local rules: (1) if a player has more resources at the end of a game than at the beginning, then the player will increase the attachment to the chosen behavior for that game. (2) If a player has less resources at the end of a game than at the beginning, then the player is going to decrease the attachment to the chosen behavior for that game. For example, if a player chose to cooperate and lost resources, then that player is going to decrease her cooperative attachment.

Players randomly choose their behavior each game by comparing the value of a random variable [0, 1] and their cooperative attachment [0.01,0.99]. If the value of the random variable is greater than the value of the cooperative attachment, the player defects. As it may be noted, the cooperative attachment can be used for labeling players as cooperators (cooperative attachment > = 0.5) or defectors (cooperative attachment < 0.5). Under certain conditions (parameter values), this model [28] exhibits an evolution of cooperation that is similar to the Kin Selection, Direct Reciprocity and Indirect Reciprocity models shown in [21].

We use two of these game-theoretic rules [28] and merge them with the lane-change freeway traffic model [29]. The merging process consists in replacing some of the abstract concepts of the game-theoretic models with information given by the freeway model: (1) Using the incentive and safety conditions we were able to create different behaviors that may be labeled as cooperatives or defectives; (2) In our model a game corresponds to the interaction between two drivers when one of them, at least, tries to perform a lane change; (3) We link the original abstract payoffs to “physical” measures that appear in the freeway traffic model dynamics. (4) We consider the two selected game-theoretic rules mentioned above as “behavior rules” and also added two more: one for describing what we think happens during a common lane change and another one considering cooperative ideas expressed in [21]. All technical aspects are detailed in the Methods section.

## Methods

**Fig 3:**
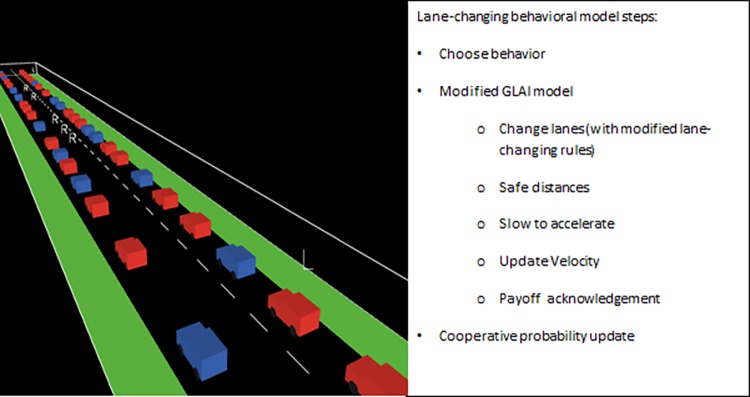
3D view (left) and steps (right) of the presented model. The model is available for running on a browser and downloading (source code included) at https://sourceforge.net/projects/traffic-games/.

The agent-based lane-changing behavioral model presented in this paper (Fig 3) can be seen as a modification of the GLAI model. Our model preserves many of the main characteristics and variables of the LAI [32] and GLAI [29] models. We made modifications to the lane-changing behavior section, *i*.*e*. instead of using probabilities to increase or decrease the lane-changing rate, the drivers increase or decrease this rate by being cooperative (more cautious incentive and safety conditions) or defective (bolder incentive and safety conditions). This means that all parameters that make simulations realistic are those that appear in the original GLAI model validation [29], except for the lane-changing probabilities to left and right and the road length. Thus, we are making the same assumptions made by the original GLAI model (safe distances between vehicles based on their relative velocities, realistic acceleration/deceleration rates and incentive and safety conditions for lane changes based on the already mentioned safe distances) with the difference that in our model the lane-changing rates are entirely a consequence of the decisions made by the drivers.

To be congruent with the GLAI model, our traffic model has a freeway defined by a lattice of length *L* with a discretization parameter (*Δx*) that indicates the cell length in meters. Each vehicle is defined by an agent that may move over the freeway cells arranged in a ring topology. Unlike the GLAI model, vehicles may move in cell fractions. The speed of each vehicle can take values from the set [0, …, *v*_*max*_], but vehicles may have different *v*_*max*_. At initialization, an average |*v*_*max*_| and a standard deviation are given as parameters to a normal distribution that randomly assign a *v*_*max*_ value to each vehicle. The *v*_*max*_ value of a vehicle may differ from |*v*_*max*_| up to four times the value of *Δv*, *i*.*e*. a vehicle’s *v*_*max*_ can take values from the set [|*v*_*max*_|–(*x Δv*)], where x ∈ [0,…,4] and *Δv* denotes the magnitude of the increase/decrease in velocity of a vehicle in one time-step under normal situations and is defined as: *Δv* = [2.5m / *Δx*]. In this model, all vehicles have the same length of 5m that also includes the space between the front bumper of the vehicle and the rear bumper of the vehicle ahead.

The vehicle density remains constant over time and every vehicle has a cooperative probability (*p*_*c*_), as in the models presented in [28]. This variable represents the drivers’ attachment to a cooperative behavior and may have a value from the set [0.1, …, 0.99]. We exclude p_c_ values 0 and 1 because they force agents to always defect and always cooperate, respectively. Such behaviors correspond to unrealistic circumstances and also lead the evolution of cooperation to dynamics that differ from those reported in [21]. All drivers with *p*_*c*_ > 0.5 are labeled as cooperators and all drivers with *p*_*c*_ < = 0.5 are labeled as defectors. This distinction among drivers is necessary because at initialization the fraction of cooperative drivers in the population is a relevant parameter. Also, *an initial-p*_*c*_*-for-cooperative-drivers* and an *initial-p*_*c*_*-for-defective-drivers* are required as two different parameters. These parameters are required because, as reported in [33], the initial values of *p*_*c*_ may have important consequences in the evolution of cooperation.

The *p*_*c*_ is a key variable in the model’s dynamic. It may be noticed that cooperative drivers do not always cooperate. Depending on their cooperative attachment, players will overcome adverse circumstances to cooperate with others. Using *p*_*c*_ and a random variable, we try to simulate this phenomenon. It is important to note that people may have different cooperative attitudes in different decision-making situations, *i*.*e*. people may have multiple *p*_*c*_ values, one for each decision-making situation. In this model we consider only lane-changing decisions.

So far we have just enumerated some similarities/dissimilarities between the original GLAI model and our model, and we have also enumerated all parameters needed. Next, we are going to detail the merging point between the GLAI model and the evolution of cooperation models and some minor changes to the original GLAI model for adapting it to an agent-based model.

### Original GLAI variables and parameters

We made an agent-based implementation of the GLAI model that preserves all aspects taken into account by the original model. GLAI parameters and variables may be found in Tables 1 and 2 respectively.

**Table 1:** GLAI and LAI model parameters.

Parameter	Description
*v*_*max*_	Maximum velocity.
*v*_*s*_	Slow velocity.
*l*_*s*_	Vehicle length (in cells).
*M*	Maximum decrease of velocity in one time step.
*R*_*s*_	Random slowing down probability.
*R*_*a*_, *R*_*0*_, *R*_*d*_	Acceleration probabilities.

Our model inherits these variables.

**Table 2:** Variables used for incentive and safety criteria in the GLAI model.

Variable	Description
*v*_*n*_	Velocity of a vehicle considering a lane change.
*d*_*n*_	Gap of the **n**-vehicle considering a lane change relative to its leader vehicle in the same lane.
*v*_*n+1*_	Velocity of the same-lane leader vehicle of vehicle *n*.
*v*_*succ*_	Velocity of the succeeding vehicle (the follower) of vehicle *n* on the target lane. The follower is the nearest vehicle on the target lane with *x* < *x*_*n*_.
*d*_*succ*_	Spatial-gap of the succeeding vehicle on the target lane relative to vehicle *n*.
*v*_*pred*_	Velocity of the preceding vehicle (possible new leader) on the target lane. The leader on the target lane is the nearest vehicle with position *x* > *x*_*n*_.
*d*_*pred*_	Gap of vehicle *n* to the preceding vehicle (possible new leader) on the target lane.
*d*_*acc*_(*v*_*n*_, *v*_*pred*_)	Safe following distance from vehicle *n* to the preceding vehicle that allows to vehicle *n* to accelerate without collision risk.
*d*_*keep*_(*v*_*n*_, *v*_*pred*_)	Safe following distance from vehicle *n* to the preceding vehicle that allows to vehicle *n* to keep its velocity without collision risk.
*d*_*dec*_(*v*_*succ*_, *v*_*n*_)	Safe following distance from the succeeding vehicle to the vehicle *n* that dictates to the succeeding vehicle to decelerate to avoid collision risks.

Our model inherits these variables.

### Modification to the original GLAI update steps

At each time step, the model updates all vehicles. An update is performed in two sub-steps, each one of them affecting all vehicles in parallel:

1.- **Exchange:** in this sub-step, the two lanes of the freeway exchange vehicles according to the lane-changing rules, which are detailed below.

2.- **Single lane update:** each of the freeway lanes is considered as independent single-lane LAI model. This sub-step operates on the resulting configuration of the exchange sub-step. For any arbitrary configuration of this sub-step, one update consists of the following (as may be seen in [32]:

**S1. Safe following distances**. Obtain the value for *d*_*dec*_ = *d*_*dec*_(*v*_*n*_(*t*), *v*_*n+1*_(*t*)), *d*_*acc*_ = *d*_*acc*_(*v*_*n*_(*t*), *v*_*n+1*_(*t*)), and *d*_*keep*_ = *d*_*keep*_(*v*_*n*_(*t*), *v*_*n+1*_(*t*)), that are the minimum required distance for a vehicle to drive at velocity *v*_*n*_ behind its preceding vehicle (*n*+1) in a safe way. The original definition of these distances can be found in [32]. Here we present modified rules that allow vehicles to move in fractions of a cell.

$$\begin{array}{l} d_{acc}(t)=[((v_{n}(t)+\Delta v)/M)+1][v_{n}(t)+\Delta v]-[M/2][((v_{n}(t)+\Delta v)/M)+1][v_{n}(t)+\Delta v)/M]-\\ \left[((v_{n+1}(t)-M)/M)+1][(v_{n+1}(t)-M)]+[M/2][((v_{n+1}(t)-M)/M)+1][(v_{n+1}(t)-M)/M\right] \end{array}$$

$$\begin{array}{l} d_{keep}(t)=[(v_{n}(t)/M)+1][v_{n}(t)]-[M/2][(v_{n}(t)/M)+1][v_{n}(t)/M]-\\ \left[((v_{n+1}(t)-M)/M)+1][(v_{n+1}(t)-M)]+[M/2][((v_{n+1}(t)-M)/M)+1][(v_{n+1}(t)-M)/M\right] \end{array}$$

$$\begin{array}{l} d_{dec}(t)=[((v_{n}(t)-\Delta v)/M)+1][v_{n}(t)-\Delta v]-[M/2][((v_{n}(t)-\Delta v)/M)+1][v_{n}(t)-\Delta v)/M]-\\ \left[((v_{n+1}(t)-M)/M)+1][(v_{n+1}(t)-M)]+[M/2][((v_{n+1}(t)-M)/M)+1][(v_{n+1}(t)-M)/M\right] \end{array}$$

The modification consists of the replacement of the function *X*_div *y*_ that denotes the integer division, *X*_div *y*_ = [*X*/*Y*], where “/” denotes normal division and [*z*] is the floor function. Instead, a normal division is indicated with “/”. This change allows one to express the safe distances in fractions but also allows negative values. To avoid negative values it is important to use the expression *d*_*x*_ = max (0, *d*_*y*_), where *d*_*y*_ represents the respective calculated safe distance (*i*.*e*. *D*_*acc*_, *D*_*keep*_ or *D*_*dec*_) and *d*_*x*_ represents the variable where the value of the respective safe distance is going to be stored. Another option is to verify the values of the safe distances during the implementation of the incentive and safety conditions. This option consists in identifying when safes distances have negative values and then handling these events.

**S2. Slow to accelerate**. This step simulates the slow reaction time of drivers. The stochastic noise parameter *R*_*a*_, dependent on the vehicle’s speed *v*_*n*_ is determined according to equation:

$$R_{a}=\min(R_{d},R_{0}+v_{n}(t)\cdot (R_{d}-R_{0})/v_{s})$$

where *v*_*s*_ is a constant parameter slightly above 0, 0 < *R*_*a*_ ≤ 1 and 0 < *R*_*0*_ < *R*_*d*_ ≤ 1. The stochastic parameter *R*_*a*_ linearly interpolates between *R*_*0*_ and *R*_*d*_ (*R*_*0*_ < *R*_*d*_) if *v*_*n*_ is smaller than a slow velocity *v*_*s*_. The stochastic parameter *R*_*a*_ indicates the probability of accelerating based on the vehicle’s velocity: slow vehicles (*v*_*n*_ < *v*_*s*_) have to wait longer before they can continue their travel.

**S3. Velocity update**. The velocity of all vehicles is updated simultaneously following the rules:
  *S3a*: *Acceleration*. If *d*_*n*_(*t*) ≥ *d*_*accn*_, the velocity of vehicle *n* increases randomly by one unit (one *Δv*) with probability (*R*_*a*_), *i*.*e*.:

$$v_{n}(t+1)=\left\{ \begin{array}{l} \min\left(v_{n}(t)+\Delta v,v_{max})\;if\;randf()\leq (R_{a}\right)\\ \;v_{v(t)\;otherwise} \end{array}\right.$$

Where *randf*() ∈ [0, 1] denotes a uniform random number and *Δv* is the maximum magnitude in cell fractions to accelerate/decelerate a vehicle under normal circumstances. It is defined as follows:

Δv=[2.5m/Δx]

*S3b*: *Random slowing down*. If *d*_*accn*_ > *d*_*n*_(*t*) ≥ *d*_*keepn*_, the velocity of vehicle *n* is decreased with a probability *R*_*s*_, *i*.*e*.:

$$v_{n}(t+1)\left\{ \begin{array}{l} \max\left(v_{n}(t)-∆v,0)\;if\;randf()\leq (Ra\right)\\ \;v_{n}(t)\;otherwise \end{array}\right.$$

*S3c*: *Braking*. If *d*_*keepn*_ > *d*_*n*_(*t*) ≥ *d*_*decn*_, and *v*_*n*_(*t*) > 0, velocity of vehicle *n* is reduced by one *Δv*:

$$v_{n}(t+1)\to \max(v_{n}(t)-\Delta v,0)$$

*S3d*: *Emergency braking*. If *v*_*n*_(*t*) > 0 and *d*_*n*_(*t*) < *d*_*decn*_(*t*), velocity of vehicle *n* is reduced by *M*, provided it does not go below zero:

$$v_{n}(t+1)\to \max(v_{n}-M,0)$$

Where *M* is the maximum decrease of velocity in one time-step.

**S4. Vehicle movement**. Each vehicle moves forward according to its new velocity defined by rules S3a-S3d:

$$x_{n}(t+1)\to x_{n}(t)+v_{n}(t+1)$$

Where *x*_*n*_(*t*) and *v*_*n*_(*t*) denote the position and the velocity, respectively, of vehicle *n* at time-step *t*.

Assuming that vehicle *n* + 1 precedes vehicle *n*, the space gap between vehicle *n* and vehicle *n* + 1 (the distance from front bumper of vehicle *n* to the rear bumper of vehicle *n* + 1) is defined as *d*_*n*_(*t*) = *x*_*n*+1_(*t*)–*x*_*n*_(*t*)–*l*_*s*_; where *l*_*s*_ denotes the vehicle length in cell fractions.

### Original GLAI lane-changing rules

These rules represent a decision that most drivers make to change lanes or not. This decision implies an incentive criterion (identify the need for a lane change), *i*.*e*. the driver expects a utility or benefit from the lane change; and a safety condition (opportunity check), *i*.*e*. in order to receive the benefit drivers must avoid collisions as a consequence of the lane change. The original GLAI rules can be seen in detail in [29] but for the convenience of the reader they are presented here as well.

right →left

Incentive criterion

$$\left(ic1):if((d_{keep}(v_{n},v_{n+1})\leq d_{n}<d_{acc}(v_{n},v_{n+1}))and(d_{pred}\geq d_{acc}(v_{n},v_{pred})and(v_{n}<v_{\max}))\right)$$

Or

$$\left(ic2):(d_{n}<d_{keep}(v_{n},v_{n+1})and(d_{pred}\geq d_{keep}(v_{n},v_{pred}))\right)$$

Safety criterion

$$\left(sc1):d_{succ}\geq d_{dec}(v_{succ},v_{n}\right)$$

left → right

Incentive criterion

$$\left(ic1'):(d_{n}\geq d_{keep}(v_{n},v_{n+1}))and(d_{pred}\geq d_{keep}(v_{n},v_{pred})\right)$$

Safety criterion

$$\left(sc1):d_{succ}\geq d_{dec}(v_{succ},v_{n}\right)$$

All variables are detailed in Table 2.

The logic behind the lane-changing rules is the following: The safety criterion (sc1) considers the effect of the lane-changing vehicle on the future follower in the target lane; if *d*_*succ*_ < *d*_*dec*_(*v*_*succ*_, *v*_*n*_) then the lane change will provoke a collision, *i*.*e*. the safety criterion guarantees that the lane change is possible regardless of whether the future follower vehicle will need to use emergency braking, normal braking, will keep its velocity, or will continue accelerating after the lane change.

The existence of two different incentive rules agrees with regulations for lane usage that apply on Mexican and European highways: One is the right-lane preference that compels drivers to use the right lane as much as possible (ic1’) in conjunction with a right-lane overtaking ban. Both the GLAI model and our agent based implementation use asymmetric rules that promote left overtaking (ic1) rather than fully banning right lane overtaking.

In the lane changes from left to right, if a vehicle is driving on the left, then as soon as possible it will attempt a change to the right lane, *i*.*e*. as soon as it’s safe following distance with respect to the vehicles ahead in both lanes is large enough to maintain its current speed.

In lane changes from right to left, if a driver wishes to improve her velocity with respect to the velocity conditions in the current lane, *i*.*e*. if the vehicle’s safe following distance to the preceding vehicle in the same lane is enough to maintain its speed, while the lane change would imply the chance to accelerate (ic1) or the vehicle’s safe following distance with respect to the preceding vehicle in the current lane implies that it will decelerate in the next time step, but on the other lane could at least maintain its current speed (ic2).

### Creation of new behaviors by modifications of the lane-changing rules

Changing the lane-changing rules results in changes in the drivers’ behavior. For example, if we replace *d*_*dec*_(*v*_*succ*_, *v*_*n*_) with *d*_*keep*_(*v*_*succ*_, *v*_*n*_) in the safety criterion (sc1), drivers will change lanes only if the succeeding vehicle in the target lane can maintain its velocity after the lane change.

New safety criterion

$$\left(sc1'):d_{succ}\geq d_{keep}(v_{succ},v_{n}\right)$$

This new behavior (in the model) may be labeled as “cautious” (as avoiding the use of braking ensures avoiding collisions provoked by the lane change). In accord with Nowak’s description, we can also describe this behavior as cooperative. If a driver finds incentive conditions, she will check the safety criterion (sc1’) to finally decide if she will change lanes or not. If *d*_*succ*_ < *d*_*keep*_(*v*_*succ*_, *v*_*n*_) then she is not going to execute the lane change. However, it may be that *d*_*succ*_ ≥ *d*_*dec*_(*v*_*succ*_, *v*_*n*_) (sc1 is satisfied), so she will be paying the cost of not improving her driving conditions and the succeeding driver will be receiving the benefit of, at least, maintaining her velocity. In general, a cooperative driver (the ones that use sc1’) will have less probability of executing a lane change, but all the succeeding drivers will have a greater probability of maintaining their driving conditions.

### Establishing the new lane-changing behavioral model

In the model we allow drivers to randomly choose between a cooperative and a defective behavior.

Cooperative right →left

Incentive criterion

$$\left(ic1):if((d_{keep}(v_{n},v_{n+1})\leq d_{n}<d_{acc}(v_{n},v_{n+1}))and(d_{pred}\geq d_{acc}(v_{n},v_{pred})and(v_{n}<v_{\max}))\right)$$

Or

$$\left(ic2):((d_{n}<d_{keep}(v_{n},v_{n+1}))and(d_{pred}\geq d_{keep}(v_{n},v_{pred}))\right)$$

Safety criterion

$$\left(sc1'):d_{succ}\geq d_{keep}(v_{succ},v_{n}\right)$$

Defective right →left

Incentive criterion

$$\left(ic1):if((d_{keep}(v_{n},v_{n+1})\leq d_{n}<d_{acc}(v_{n},v_{n+1}))and(d_{pred}\geq d_{acc}(v_{n},v_{pred})and(v_{n}<v_{\max}))\right)$$

Or

$$\left(ic2):(d_{n}<d_{keep}(v_{n},v_{n+1})and(d_{pred}\geq d_{keep}(v_{n},v_{pred}))\right)$$

Safety criterion

$$\left(sc1):d_{succ}\geq d_{dec}(v_{succ},v_{n}\right)$$

The incentive criterion for right → left lane change is the same for both cases (cooperative and defective) and is the same incentive criterion as for right → left lane changes in the GLAI model. The only change between cooperative and defective behavior is that cooperators use the second version of the safety criterion (sc1’) and defectors use the original version. The logic behind this, as mentioned earlier, is that cooperative drivers will pay the cost of having less probability of changing lane for the succeeding vehicles will have a higher probability of maintaining their driving conditions, while defective drivers will take any opportunity of improving their driving conditions regardless of the consequences for other drivers (as long as collisions are avoided).

As we are working with a model that is in accord with the regulations used on Mexican and European highways, we have the corresponding asymmetric lane changes rules:

Cooperative left → right

Incentive criterion

$$\left(ic1'):(d_{n}\geq d_{keep}(v_{n},v_{n+1}))and(d_{pred}\geq d_{keep}(v_{n},v_{pred})\right)$$

Safety criterion

$$\left(sc1'):d_{succ}\geq d_{keep}(v_{succ},v_{n}\right)$$

Defective left →right

Incentive criterion

$$\left(ic1):if((d_{keep}(v_{n},v_{n+1})\leq d_{n}<d_{acc}(v_{n},v_{n+1}))and(d_{pred}\geq d_{acc}(v_{n},v_{pred})and(v_{n},v_{\max}))\right)$$

Or

$$\left(ic2):(d_{n}<d_{keep}(v_{n},v_{n+1})and(d_{pred}\geq d_{keep}(v_{n},v_{pred}))\right)$$

Safety criterion

$$\left(sc1):d_{succ}\geq d_{dec}(v_{succ},v_{n}\right)$$

The cooperative criteria almost agree with the original left →right criteria of the GLAI model, but we make it more cautious, *i*.*e*. cooperative drivers are going to return to the right lane as soon as possible (ic1’), ensuring thereby not to alter the driving conditions of the succeeding vehicle in the target lane (sc1’). Defective drivers, on the other hand, will use the left lane as much as they want (ic1’ is omitted as part of the incentive conditions), they will be more likely to use the right lane to overtake (ic1 and ic2), and they only will avoid collisions as a consequence of the lane change (sc1).

In real life, there are other actions that may be labeled as cooperative or defective. We consider two such actions to better model the consequences for being defective or cooperative.

**Directional signals**: in most countries, drivers are required to use directional signals to indicate the intention of changing lanes. In Mexico and other countries this is commonly disobeyed, especially during traffic jams where the use of directional signals carries adverse consequences, as drivers may try to obstruct others from changing lane. To simulate these actions we give the drivers the ability to choose between displaying directional signals (cooperative) or not (defective). Right after checking the incentive criterion, if the driver has chosen to cooperate and the incentive criterion is satisfied, then the driver will display directional signals indicating her intention to change lane. Right after the driver checks the safety criterion: if it is satisfied then the lane change will take place, but if the safety criterion is not satisfied then the driver will keep the directional signal activated and continue normally with the rest of the sub-steps. The only cases in which a driver will turn off the directional signal are: (1) a subsequent incentive criterion is not satisfied; and (2) the lane change was successfully executed.

**Facilitate lane changes**: when a driver activates her directional signal, it is supposed that the future succeeding driver in the target lane will acknowledge the first driver’s intentions and will take precautionary measures if needed, *i*.*e*., if the future succeeding driver foresees that the lane change will take place then a precautionary measure could be to start generating a safe distance for the future preceding vehicle. This can be achieved by not accelerating or by gradually slowing down. Frequently in Mexico, when a driver acknowledges the lane-change intentions of other drivers she doesn’t facilitate the lane change and maintains her velocity or even accelerates.

We model these lane-changing behaviors by letting the drivers randomly choose, with the help of *p*_*c*_, between facilitating the lane change (cooperative) or continue driving, ignoring the lane change intentions of the others (defective). When a driver cooperatively attempts to change lane, the future succeeding driver in the target lane (target vehicle) decides if she will have a cooperative or a defective behavior. If she decides to be cooperative, she tries to facilitate the lane change by not accelerating or by braking if needed. To do this we have modified the S3c conditions of the Velocity update section of the Single lane update sub-step:

S3c’: Braking’.

If [(*cooperative-target*? = = true and *directional-signals-detected*? = = true) and (*d*_*keep-pred’*_ > *d*_*pred’*_(t) ≥ *d*_*dec-pred’*_, and *v*_*n*_(*t*) > 0)]

or [(*d*_*keepn*_ > *d*_*n*_(*t*) ≥ *d*_*decn*_,) and (*v*_*n*_(*t*) > 0)], velocity of vehicle *n* is reduced by one *Δv*:

$$v_{n}(t+1)\to \max(v_{n}(t)-\Delta v,0)$$

Where *cooperative-target*? is a tag indicating that driver *n* chose to be cooperative and she could be the succeeding driver of another driver with lane-change intentions; *directional-signals-detected*? indicates that the driver with lane-change intentions is a cooperator and that her directional signal is on; *d*_*pred’*_*(t)* is the spatial gap of target vehicle *n* to the preceding vehicle (possible new leader) on the same lane of vehicle *n; and d*_*keep-pred’*_ is the safe following distance from vehicle *n* to the preceding vehicle (possible new leader) that allows vehicle *n* to keep its velocity without collision risk.

This covers the cases in which the target driver slows down in order to facilitate the lane change, but where, in many cases, to stop accelerating is enough. To model this, we have modified the S3a conditions of the Velocity update section of the Single lane update sub-step:

S3a’: Acceleration’.

If (*target*? = = false or *cooperative-target*? = = false or (*cooperative-target*? = = true and *directional-signals-detected*? = = false))

and (*d*_*n*_(*t*) ≥ *d*_*accn*_), the velocity of vehicle *n* increases randomly by one unit (one *Δv*) with probability (*R*_*a*_), *i*.*e*.:

$$v_{n}(t+1)=\left\{ \begin{array}{l} \min\left(v_{n}(t)+\Delta v,v_{max})\;if\;randf()\leq (R_{a}\right)\\ \;v_{v(t)\;otherwise} \end{array}\right.$$

The first part of the conditional indicates the cases in which the drivers will accelerate as usual: *target*? = = false indicates that drivers in front of driver *n* on the other lane don’t have lane-change intentions; *cooperative-target*? = = false indicates that driver *n* is the target future succeeding vehicle of another driver but driver *n* has chosen to be defective; and, finally, (*cooperative-target*? = = true and *directional-signals-detected*? = = false) indicates that driver *n* is participating in a lane change, driver *n* has chosen to be cooperative, but as the lane changing vehicle has chosen to be defective (not using her directional signals) driver *n* was unable to react properly. As a result of the exclusion of the (*cooperative-target*? = = true and *directional-signals-detected*? = = true) case, when these conditions are met the driver will not accelerate.

### Evolution of cooperation dynamics

As in previous work [28], each agent has a cooperative probability that defines their attachment to a cooperative behavior. At the beginning of a lane-change decision (right before checking the incentive criterion), each driver decides if she is going to exhibit a cooperative or a defective behavior according to:

$${behavior}_{n}=\left\{ \begin{array}{l} \mathrm{cooperative}\;if\;randf()\leq p_{cn}\\ defective\;if\;randf()\;>p_{cn} \end{array}\right.$$

Where *randf*( ) ∈ [0,1] denotes a uniform random number.

In the model implementation, once an active driver finds incentive conditions to change lane, she will track down the corresponding probable future-succeeding driver (target driver) and mark her as a game partner. Once a target driver is marked as a game partner, she will decide which behavior to exhibit. Note that an active driver may have only one game partner but target drivers could have more than one game partner. If a target driver is marked by multiple active drivers, she only will decide which behavior to exhibit once and will keep that decision through the complete time-step.

**Fig 4:**
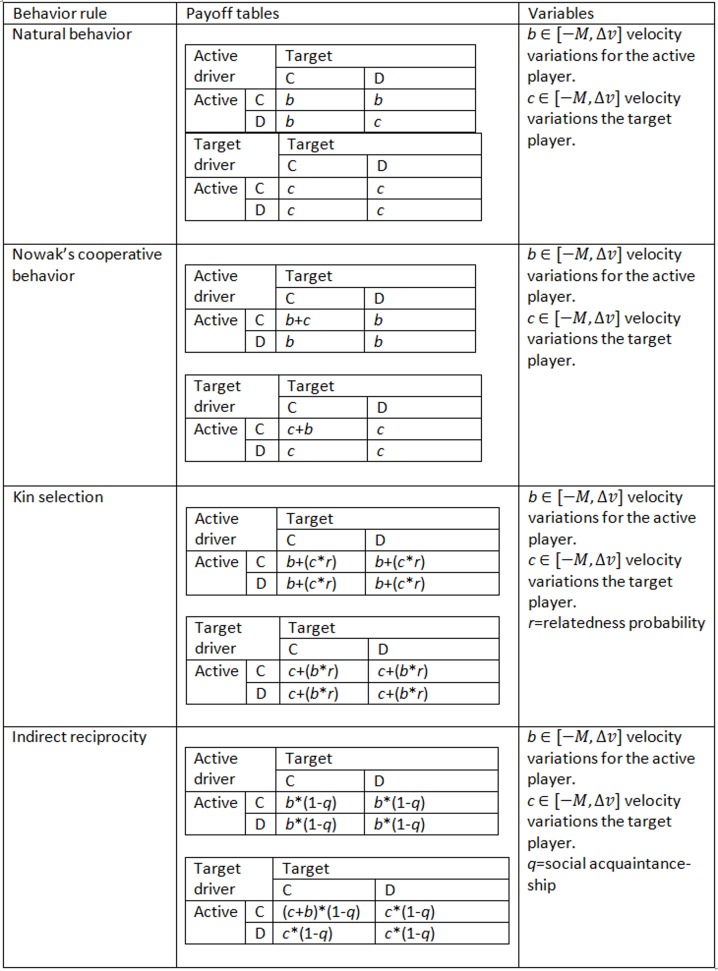
Cost-benefit notions (behavior rules) described as payoff matrixes specifying the interactions between drivers.

Once the drivers have decided which behavior to exhibit, the steps of the GLAI model are executed with the modifications already mentioned. Right before the “vehicle movement section” of the single lane update sub-step, each driver will acknowledge the consequences of their decisions by recognizing the costs paid and the benefits gained. In general, drivers will consider each loss of velocity as a cost and every velocity increase as a benefit. Nevertheless, we implemented different cost-benefit notions to study the impact of different behaviors (cooperative/defective) in conjunction with different cost-benefit notions (behavior rules). These rules let us easily add different elements to the bounded rationality of the agents by modifying the pay-off tables and without the need of making major modifications to the model dynamics. For implementation, it is required to introduce a parameter to choose between the rules. We create the following rule cases (Fig 4):

**Natural behavior:** with this case we represent what seems to occur during lane changes. In the cases where the active driver accomplishes the lane change, she always receives a benefit, given the nature of the incentive criterion (lane change to improve or at least maintain driving velocity conditions). Given the different conditions present in the freeway (different densities) and the different behaviors of target drivers, they may continue accelerating, maintain velocity, slow down or use emergency braking.

In other cases the active driver will not be able to accomplish the lane change. In these cases the active driver may continue accelerating, maintain her velocity, slow down or use emergency braking. In the cases in which the target driver is a defective driver, the chances for the active driver to slow down or use emergency braking will increase.

In a strict sense, the natural behavior payoff table only reports to the driver the gains or losses that she gets as a consequence of her participation in a lane change, which is used to change her cooperation probability in further lane change attempts.

**Nowak’s cooperative behavior:** this is a modified version of the natural behavior. In all of the rules for the five mechanisms for the evolution of cooperation studied by Nowak [21], for the cases in which both players cooperate, the expression *b-c* implies that the impact of the benefit and the cost affect them. This could represent, in lane changes, empathy between drivers. A target driver cooperating could compensate her velocity decrease with an emotional reward and in the same way as a cooperative active driver could not completely enjoy her velocity increase due to a negative emotion. We cannot confirm whether this happens during actual lane changes, especially the last situation mentioned, but we included this behavior in this study in order to explore the consequences of this behavior.

**Kin selection:** we model this case because it may represent some actual driving circumstances. In [21], kin selection is explained as the manner in which natural selection favors cooperation. While the relatedness probability (probability of sharing genes) increases, so does the probability of cooperating. While driving, this may be understood as a circumstance in which the other driver participating in the lane change is related to us. A genetic relation is not necessary, it is sufficient that drivers are acquainted (such as a coworker [including professional drivers], neighbor, friend, *etc*). Each case will have a different relatedness value according to how kindred each agent is to the other driver.

It is important to mention that the model supposes a uniform relatedness probability among all drivers. Also, we did not consider cases where recognition could actually trigger an aggressive behavior, *e*.*g*. between taxi and Uber drivers.

**Indirect reciprocity:** as in the models of [21] and [28], reputation (social acquaintanceship of our actions) is the key feature for the evolution of cooperation. As may be seen in the corresponding row of Fig 4, reputation will compensate cooperative behaviors and will punish defective behaviors.

As in the models of [21] and [28], cooperative drivers only cooperate when they acknowledge another cooperative driver. Drivers will have a chance to recognize the other driver as a defector, if *randf*() < = *q*, and then exhibit a defective behavior with that driver. If *randf*() > *q*, the driver fails to recognize the defective driver and will exhibit a cooperative behavior.

### Cooperative probability update

Finally, after estimating the consequences of their decisions (payoff of the last game played); drivers decide whether they maintain, increase, or decrease their attachment to a cooperative or defective behavior. Like the agents of the models presented in [28], drivers update their *p*_*c*_ using the expression:

$$p_{cn}=p_{cn}+\Delta p_{cn}$$

Where *Δp*_*cn*_ is calculated as follows:

$$\Delta p_{cn}\left\{ \begin{array}{l} +0.01\;if\;\left(behavior_{n}==cooperative\right)and\;(payoff_{n}\left(t-1\right)<payoff_{n}\left(t\right))\\ +0.01\;if\;\left(behavior_{n}==defective\right)and\;(payoff_{n}\left(t-1\right)>payoff_{n}\left(t\right))\\ -0.01\;if\;\left(behavior_{n}==defective\right)and\;(payoff_{n}\left(t-1\right)<payoff_{n}\left(t\right))\\ -0.01\;if\;\left(behavior_{n}==cooperative\right)and\;(payoff_{n}\left(t-1\right)>payoff_{n}\left(t\right))\\ \;0\;otherwise \end{array}\right.$$

where *payoff*_*n*_(*t*-1) denotes the payoff calculated in the time-step previous (*t*-1) to the actual time-step (*t*) following the corresponding payoff (Fig 4) for driver *n* and *payoff*_*n*_(*t*) represents the payoff calculated in the actual time-step (*t*).

After an update, each driver must verify that she doesn’t reach an invalid *p*_*c*_ value:

            if (*p*_*c*_ < = 0.00){

                    *p*_*c*_ = 0.01

            }

            else{

                    if (*p*_*c*_ > = 1) {

                            *p*_*c*_ = 0.99

                    }

            }

This also helps us to simulate the fact that even extremely attached cooperators or defectors may have situations in which they will choose actions opposed to their beliefs, *e*.*g*. by distraction.

### Parameter values used in our analysis

Since experimental data for cooperative rates is lacking, we simply swept the parameter space to understand the potential dynamics. All results were obtained by averaging the outcome of 10 simulations with the same initial parameter values. Standard deviations were so low (maximum 0.02%) that they were omitted. Each of these 10 outcomes consists of calculating the average of the last 10,000 values of the observed variables. For each simulation, 30,000 iterations were executed prior to the observation in order to avoid measuring transient states. Each iteration represents one second, thus the observation period has a duration of 2.7 hrs and was preceded by an 8.3 hrs period for relaxation of the system.

For the GLAI section of the model, we used the following parameter values: L = 120 (cells), *Δx* = 5.0m (resulting in a two-laned one-way freeway with cyclic boundaries and a length of 600m for each lane), *car size* = 5m, *R*_*d*_ = 1.00, *R*_*0*_ = 0.8, *R*_*s*_ = 0.01. We did not consider larger lane lengths due to the games used in drivers’ interactions, in particular, Kin Selection and Indirect Reciprocity. Even though it is possible to implement these games on longer freeways, it is difficult to imagine that drivers at the beginning of a freeway are aware of the behavior of other drivers that are located more than 5 km away (in this paper we are not considering V2V communication scenarios). Also, we performed simulations with L = 120, 240 and 300 cells that showed no significant differences in terms of traffic performance and in terms of the evolution of cooperation.

For *density*, we explored the following values: 0.01, 0.02, 0.03, 0.04, 0.05, 0.06, 0.07, 0.08, 0.09, 0.10, 0.11, 0.12, 0.13, 0.14, 0.15, 0.16, 0.17, 0.18, 0.19, 0.20, 0.21, 0.22, 0.23, 0.24, 0.25, 0.26, 0.27, 0.28, 0.29, 0.30, 0.35, 0.40, 0.45, 0.50 and 0.60. These values correspond in veh/km to: 2, 4, 6, 8, 10, 12, 14, 16, 18, 20, 22, 24, 26, 28, 30, 32, 34, 36, 38, 40, 42, 44, 46, 48, 50, 52, 54, 56, 58, 60, 70, 80, 90, 100 and 120. All vehicles were placed randomly along the freeway avoiding collisions (fractions of two different vehicles occupying the same space). For the implementation of heterogeneously maximum velocities we used a Gaussian distribution with a *standard deviation* of 0.7 and a |*v*_*max*_| = 6 cells/tick (equivalent to 30 m/s or 108 km/hr). In order to avoid collisions, all vehicles start the simulation with *v* = 0 cells/tick.

For our first evolution of cooperation description, we want to define the impact of the initial fraction of cooperative drivers. We used an *initial-p*_*c*_*-for-cooperative-drivers* = 0.99, *initial-p*_*c*_*-for-defective-drivers* = 0.45 and we varied the *initial–fraction-of-cooperative-drivers* with the values: 0%, 25%, 50%, 75%, and 100%. The values for the initial cooperative probabilities were chosen taking into account the results showed in [33].

Once we found that the evolution of cooperation follows the same pattern regardless of the initial fraction of cooperators, in the following, we used the same initial cooperative probabilities values already mentioned and an *initial–fraction-of-cooperative-drivers* = 50%.

For the study of the impact of the feature exploited by the behavior rules (Kin Selection and Indirect Reciprocity) we obtained the evolution of cooperation for different values for *r* and *q* respectively. The values explored were: 0.25, 0.5, 0.75 and 1.

### Mobility index

We introduce a mobility index calculated by the following expression:

$$f(td,st,t,ted,ts,v)=\left\{ \begin{array}{l} tanh(2.5(\frac{\left(td-st)-(\left(\frac{t-ted}{v})+(ts-st\right)\right)}{td-st})),\;v>0\\ \;tanh(2.25(\frac{\left(td-st)-(ts-st\right)}{td-st})(\frac{t-ted}{t})),\;v=0 \end{array}\right.$$

We assume that drivers have a starting and a destination point. Thus, they have a distance to travel (*t*) and they have a time deadline (*td*) to cover that distance. For the experiments we used *t* = 1.2 km and *td* = 500 s. Using the instant velocity (*v*) of a vehicle at a given moment and the already traveled distance (*ted*), we calculate how many time steps the driver will need to complete her travel. We add that expected time to the current time step (*ts*) to obtain an expected total travel time. We then compare the expected total time with the time deadline and finally we obtain the proportion of that difference regarding the time deadline.

When a driver finishes a trip, she immediately starts a new one (1.2 km). To maintain the time variables relative to the current trip, we take into account the starting time step (*st*) of each trip.

All calculations mentioned above are valid when *v*>0 but a zero division will arise when *v* = 0. To overcome this, we use a different expression for a stopped vehicle. We start by checking for the difference between the time deadline and the time already consumed by the driver, the proportion between this difference and the time deadline is obtained and that will give us a notion of how much time the driver has left. As time passes this factor will decrease. Finally, the decreasing factor is multiplied by the proportion of the travel that has already been completed.

In both cases, the resulting value is evaluated using the tanh(*x*) function to obtain a sigmoidal characterization and the 2.5 coefficient is utilized to fix the graphic in the [–1,1] interval, being *mobility* = 1 circumstances in which the driver arrived early at her destination, *mobility* = 0 for circumstances in which the driver arrived just in time to her destination and *mobility* = -1 circumstances in which the driver arrived late to her destination.

## Results

We performed a series of simulations to study the evolution of cooperation in a game-theoretic/agent framework and the corresponding traffic performance. As we lack real data for which fractions of drivers exhibit cooperative or selfish (expected to vary across cities), and considering that in practice these probabilities can vary contextually for the same drivers, we swept the parameter space to explore all possibilities. Changes are gradual, but it is easier to understand extreme cases, and these are the ones we focus on. For traffic performance comparison we aggregate the results obtained by an original GLAI model implementation [29] which was inspired by observational studies [32].

**Fig 5:**
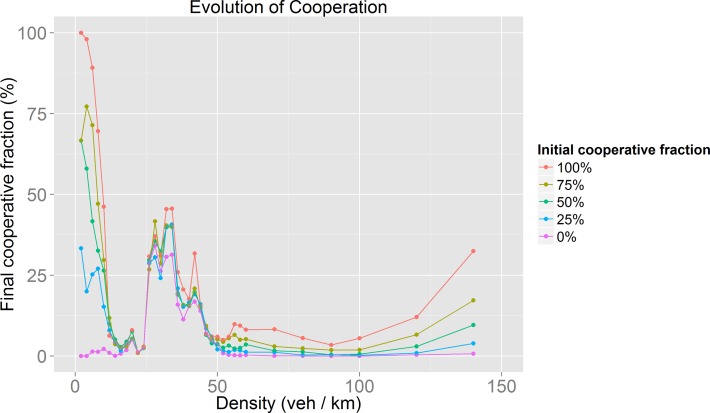
Results of the evolution of cooperation for different densities and different initial fractions of cooperative drivers.

As can be seen in Fig 5, we found that the evolution of cooperation follows the same pattern regardless of the initial fraction of cooperative drivers. As a next phase, we analyzed the impact of the feature exploited by the behavior rules that, in our opinion, could be better adapted to real traffic situations: Kin Selection and Indirect Reciprocity. For these we explored different values (0.25, 0.5, 0.75 and 1) for *r* (relatedness probability for Kin Selection) and *q* (social acquaintanceship for Indirect Reciprocity).

**Fig 6:**
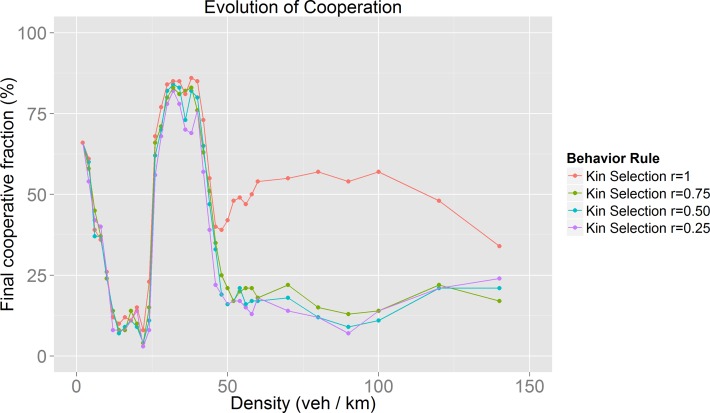
Kin Selection evolution of cooperation as density and relatedness probability are increased.

**Fig 7:**
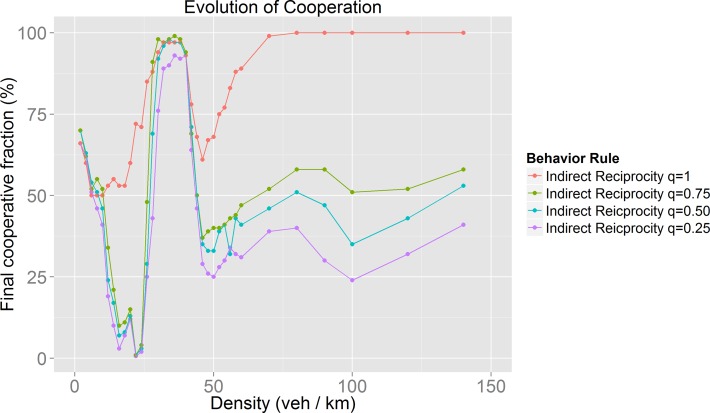
Indirect Reciprocity evolution of cooperation as density and social acquaintanceship are increased.

**Fig 8:**
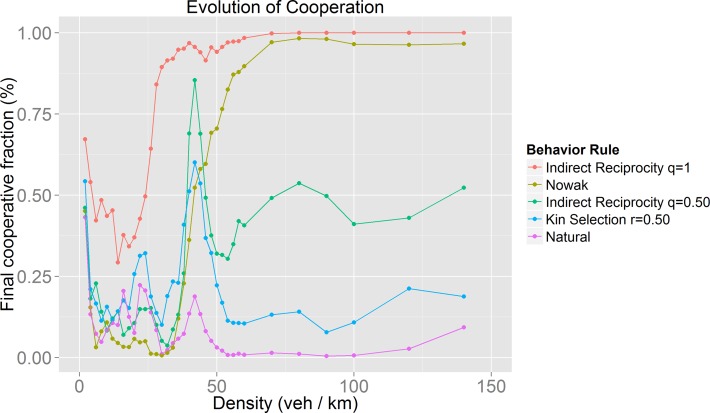
Comparison between different behaviors. As explained in the Evolution of Cooperation subsection of Methods, each behavior corresponds to a different payoff matrix.

As may be observed in Figs 5–8, there is a recognizable evolution of cooperation pattern for densities in which synchronized flow occurs. Because of the use of an evolution of cooperation approach, we expected to find conditions in which cooperation prevails. Even though the behavior of the found pattern differs from our expectations, we found it interesting. It suggests that there are conditions for driver’s self-organization to achieve the evolution of cooperation and to optimize mobility despite the greedy nature of the lane-changing rules and the payoff tables that drivers are following at a local level (drivers seek to obtain more velocity or to maintain it, they are “rewarded” for doing so and are “punished” for slowing down). As an attempt to explore this phenomenon, we obtained the average velocity for the empirical behavior with 50% of initial cooperators and using the parameters already mentioned.

**Fig 9:**
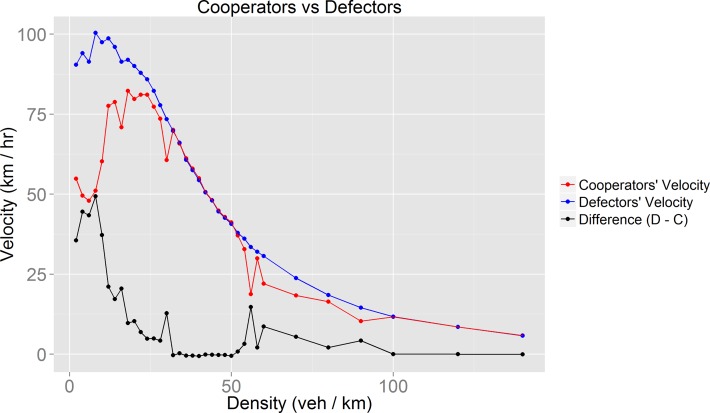
Velocities of cooperative and defective drivers.

**Fig 10:**
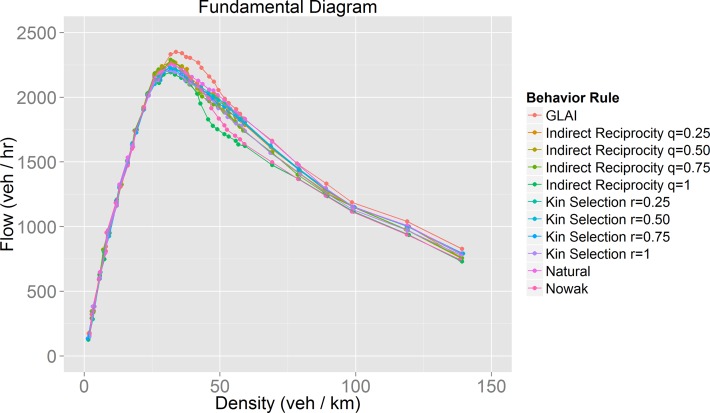
Fundamental diagram for each of the behaviors studied. A detailed view of the synchronized flow phase can be seen in Fig 11.

**Fig 11:**
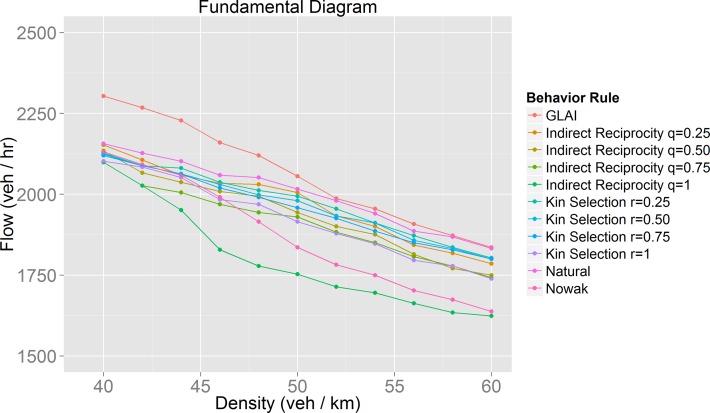
Detailed view of synchronized flow phase of the fundamental diagram (Fig 10).

If we focus on Fig 5 and Fig 9, it is noticeable that as the difference in velocity between defectors and cooperators decreases, the fraction of cooperators among the population increases. This inverse proportionality is well illustrated by the significant cooperators velocity decrease for densities around 34 veh/km. This fall causes a big difference between the velocity of defectors and cooperators which leads to a fall in the population’s cooperative fraction. We find it interesting that this proportionality is only valid for density values in which synchronized flow occurs (Fig 10 and Fig 11).

At densities between 10 and 20 veh/km we observed a decrease in cooperators velocity. This decrease is due to many factors: First, at low densities there are few cooperative vehicles, thus velocity variations are more significant; another factor is that vehicles do not have the same maximum velocity (*v*_*max*_). The lowest possible value is *v*_*max*_ = 81km/hr. Also, while facilitating lane changes, cooperators slow down (we observed that vehicles may slow down up to 45 km/hr while facilitating lane changes).

For densities near 40 veh/km in Fig 7 it could be possible to identify coherent moving state conditions [34]: cooperators and defectors have the same velocity despite the fact that vehicles of both groups have heterogeneous *v*_*max*_ values.

In order to verify that a coherent moving state is occurring, we evaluated our proposed mobility index. The values of this index are in the interval [–1, 1], with *mobility* = 1 signifying circumstances in which the driver arrived early at her destination, *mobility* = 0 for circumstances in which the driver arrived in time to her destination, and *mobility* = -1, signifying circumstances in which the driver arrived late to her destination.

**Fig 12:**
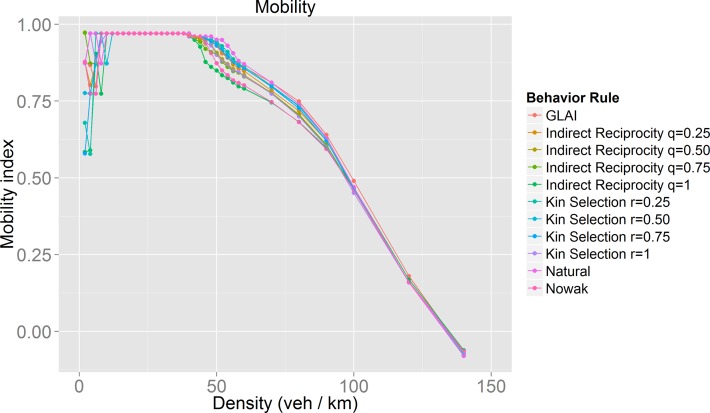
Results for the mobility index for each of the behaviors studied. A detailed view of the synchronized flow phase can be seen in Fig 13.

**Fig 13:**
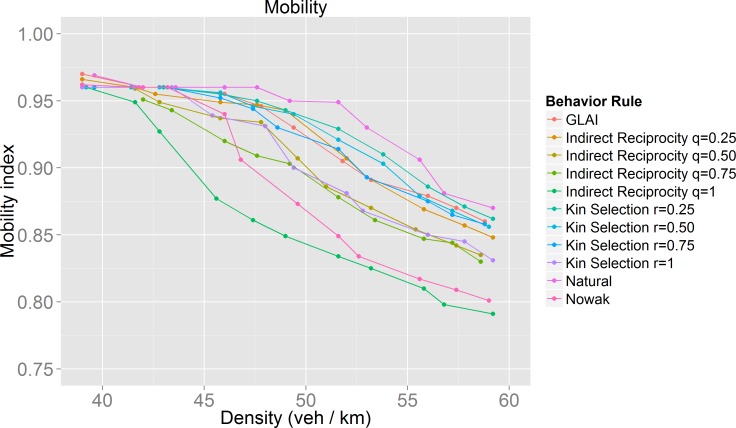
Detailed view of the mobility index in the synchronized flow phase for the different behaviors (Fig 12).

Fig 12 and Fig 13 show that at densities for which cooperators and defectors have the same velocity (Fig 9), drivers indeed have the best mobility index confirming the maximization nature of the game performed by the drivers.

**Fig 14:**
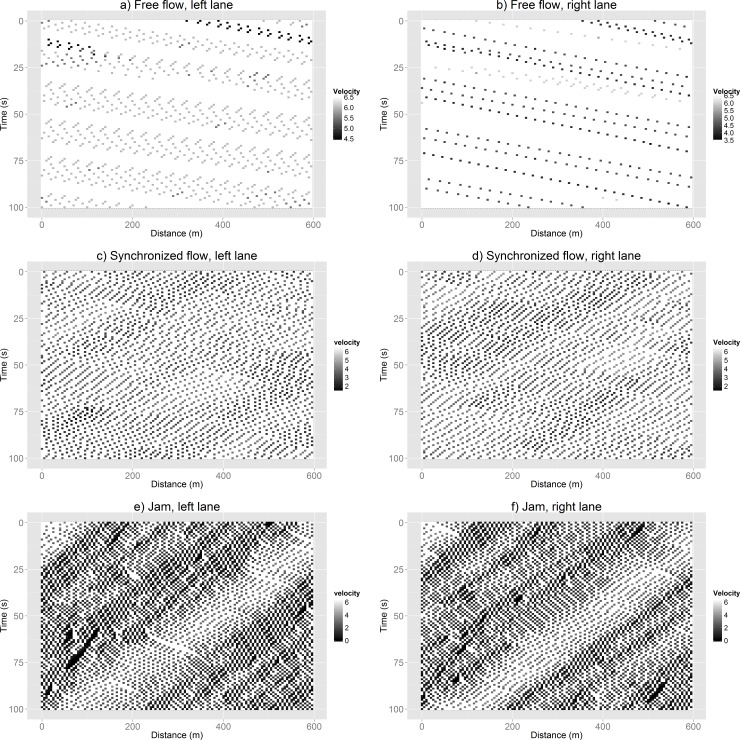
Spatiotemporal shapes of the proposed model with the behavior that presents improved mobility. (a, b) Free flow, *d* (density) = 10 veh/km, (c, d) synchronized flow, *d* = 40 veh/km (e, f) and jammed *d* = 80 veh/km. The left (right) image corresponds to the left (right) lane.

Trying to confirm the coherent movement state, we explore the spatiotemporal shapes of the original GLAI model and different behaviors implemented in our model with different density values. In Fig 14 we present the different states of traffic flow (free flow, synchronized flow and wide moving jam) for our model with “Natural” behavior. Each horizontal row of squares represents the instantaneous position of the vehicles moving towards the right. Successive rows represent the position of the same vehicles at successive time-steps. Darker squares represent vehicles closer to *v* = 0.

**Fig 15:**
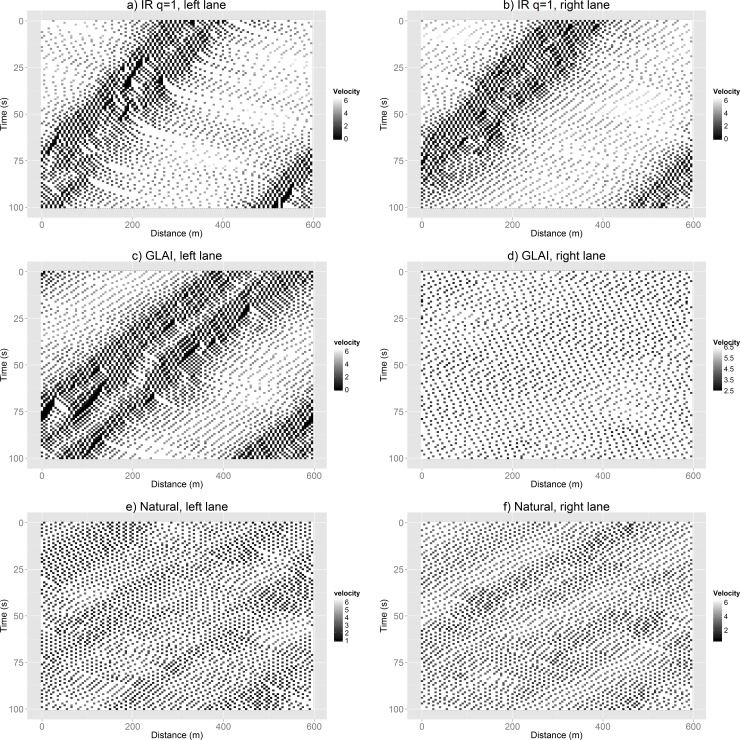
Spatiotemporal shapes for (a, b) Indirect Reciprocity with *q* = 1 (highly cooperative lowest mobility behavior), (c, d) original GLAI model (stochastic middle mobility behavior) and (e, f) the behavior we labeled as “Natural” (highly adaptive, highest mobility behavior) for the same density values (*d* = 50 veh/km). The left (right) image corresponds to the left (right) lane.

In Fig 15 we present the spatiotemporal shapes for a high cooperation behavior (Indirect Reciprocity with *q* = 1), the original GLAI model behavior and the behavior with improved mobility (the one we labeled as “Natural”) for the same density value (*d* = 50 veh/km). It may be appreciated that the high cooperation behavior (the one with worst mobility index in Fig 12) presents both lanes in jammed conditions (Fig 15A and 15B). The original GLAI model behavior (average mobility index in Fig 12) presents its left lane with jammed conditions (Fig 15(C)) and its right lane is in synchronized flow (Fig 15(D)). While the behavior that we labeled as “Natural” (the one with best mobility index in Fig 12) presents both lanes in conditions that may be identified as a transition between the synchronized phase and the jammed phase (Fig 15E and 15F). It is worthy to mention that this conditions (Fig 15E and 15F) represent better traffic performance that those obtained by the other two behaviors (Fig 15A–15D). The results reflected in the spatio-temporal shapes agreed with the results shown in the fundamental diagrams of Fig 10.

**Fig 16:**
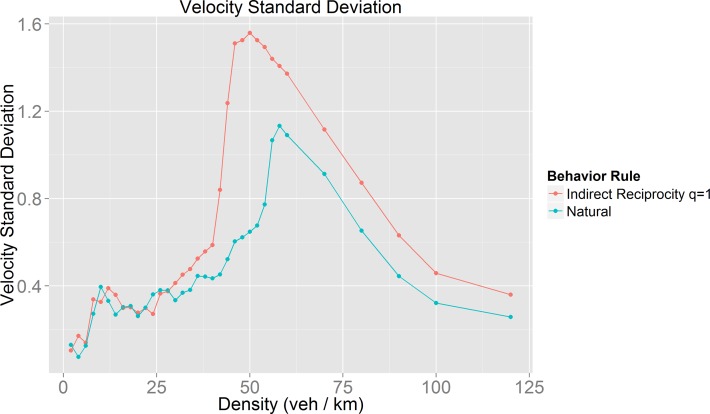
Mean velocity standard deviation obtained to every density value studied for two different behavior rules, Indirect Reciprocity with q = 1 (high cooperation behavior) and Natural (behavior with improved mobility).

In order to prove or disprove the existence of a coherent movement state we obtain the average standard deviation of the average velocity of all vehicles for each studied density value for the high cooperation behavior (Indirect Reciprocity with q = 1) and the behavior with the improved mobility (labeled as “Natural”) (Fig 16). For density values corresponding to the beginning of the synchronized phase, the graphic shows values very similar to those found in the free flow phase. As density increases the standard deviation, values increase as well. This means that, as the fundamental diagram implies, the synchronization between vehicles’ velocity start to fade as the density values approach to the jammed phase.

The results in Fig 16 show that, once the synchronized phase starts, the high cooperation behavior has a larger standard deviation than the behavior with improved mobility and this characteristic remains even during the jammed phase. The effect of the Natural behavior rules over the vehicles’ velocity standard deviation has a notorious impact, precisely, at the end of the synchronized phase (density values between 40 and 50 veh/km) where the difference between vehicles’ velocity standard deviation of the two behavior rules is about twice. This proves that, the Natural behavior rule favors the emergence of a coherent movement state by reducing the vehicles’ velocity standard deviation and thus improving mobility.

**Fig 17:**
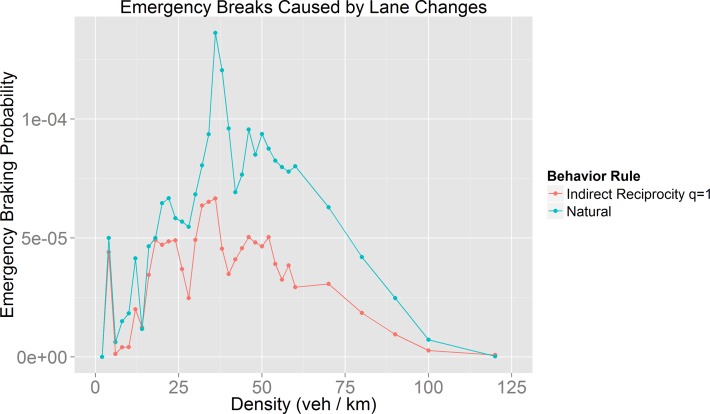
Probability of emergency brakes caused by lane changes for Indirect Reciprocity with *q* = 1 and Natural behaviors.

In a different matter, many traffic regulations promote safe behaviors (*e*.*g*. use of directional signals and facilitate lane changes for vehicles with directional signals). As we include some of these behaviors (labeled as cooperative behaviors) we expect that behavior models with high cooperation rates (*e*.*g*. Indirect Reciprocity with *q* = 1) present better safety conditions. As there are no accidents in our model, in order to measure the safety of different driving behaviors, we calculated the probability of emergency brakes caused by a lane change. The rationale is that more emergency breaks increase accident incidence. Fig 17 shows this probability for Indirect Reciprocity with *q* = 1 (more cooperators) and for Natural (less cooperators).

The results show that, even though, the Natural behavior rule has an improved mobility (thanks to the reduced vehicles’ velocity standard deviation) it has a bigger emergency breaking probability and thus it does not guarantee safer driving conditions. The behavior that produces safer driving conditions (lower emergency braking probability) is the high cooperation behavior (Indirect Reciprocity with q = 1), because most of the drivers exhibit a cooperative behavior and they do not change lane if they are going to cause other drivers to break.

## Discussion

### Model Novelty

In this paper a novel approach for studying freeway traffic was presented. This game theoretic approach allowed us to model different behaviors using well-studied games and rules [21]. By merging these games and rules with an agent based implementation of a realistic freeway traffic model [29], we were able to categorize agents (drivers) into two groups (cooperators and defectors), assign them characteristic behaviors and let them choose which behavior they want to exhibit more frequently to maximize their individual performance. We consider it relevant to remark that this type of model combination may be useful for other spatial systems. The process implies a detailed understanding of the phenomena to be modeled, to identify behaviors that may have an impact on the system’s performance and values generated by the system that may be used as payoffs for the games.

In the model that we studied, freeway lane changing interactions, the lane-changing rules were modified to create different behaviors. This was because the rules were based on distance and velocity and the payoff tables make use of drivers’ velocities values.

Merging game-theoretic models with spatial models may not be trivial, but given the decision-making formal description of game theory, the resulting model provides relevant information about the interactions among the agents. This information can lead to a better understanding of the phenomena studied.

We consider this approach to have applications to systems with a strong human component, such as pedestrian movement or social networks.

### Evolution of cooperation

The results of our model for the evolution of cooperation (Fig 5 to Fig 8) show that cooperative behavior may succeed only for certain values of the traffic density. It is worth noting that for many of the behaviors modeled these density values correspond to those in which the synchronized flow phase takes place (Fig 10). It is important to remember that unlike other models [11], the cooperative or politeness degree exhibited by the drivers is internally and individually chosen by the agents themselves. There is no global variable, nor parameter, to guide their behavior. The coincidence between the large increase in the fraction of cooperators for certain density values implies that only during the synchronized phase are conditions for drivers’ self-organization present. Despite the selfish nature of the payoff matrices (drivers are rewarded by increasing their speed and punished by slowing down) the drivers found a circumstance in which not pursuing a velocity increase (cooperative behavior) ended up rewarding them. This can be seen as an example of the slower-is-faster effect [35].

There are behaviors (Kin Selection, Indirect Reciprocity and Nowak’s Cooperation) in which the evolution of cooperation also occurs during the jammed phase. The evolution of cooperation results reflect the fact that the map between driver environment and driver behavior is complex, as different environments may lead to the same global behaviors, while different perceptions of drivers may produce different global behaviors.

We can explain this evolution of cooperation behavior occurring in the synchronized phase and in the moving jam phase as being due to the fact that these phases are highly related to the amount of interactions (lane changes) occurring for each density value. In the moving jam phase there are few spaces that may be used by drivers to perform lane-changing maneuvers, leading to less interaction between drivers and reducing the effect of cooperative or defective lane changes. Free flow conditions also lead to low levels of interactions due to large spaces between vehicles. These large spaces allow agents to use the road more freely, i.e. without taking into account the behavior of other drivers. Also, these large spaces prevent the propagation of cost (*c*) among vehicles. During the synchronized phase the cost paid by a target vehicle (vehicle that will became the new follower of a lane changing vehicle), probably, is going to be propagated among many vehicles. This happens when a lane changing vehicle forces the target vehicle to brake and, due to the spaces between vehicles in the target lane, many other vehicles will brake too.

Taking into account the previous reasoning, we argue that traffic phases have more impact on the evolution of cooperation than the other way around. In the particular case of the synchronized phase, it is because of the amount of vehicles and the amount of free space that drivers increase their lane-changing rate. And it is this increase that generates the promotion of cooperation. At any density, a particular target vehicle (vehicle that will became the new follower of a lane-changing vehicle) may obtain four possible payoffs: a decrease of 5 m/s (emergency braking), a decrease of 2.5 m/s (normal braking), neither increase nor decrease (lane change with enough space or lane change not executed) or an increase of 2.5 m/s (lane changing with enough space or lane change not executed). Velocity increases will promote the action (cooperate or defect) while the decreases are responsible for the promotion of cooperative behavior. A cooperative target driver will stop accelerating or even braking to help the other driver to execute the lane change maneuver. This means that if she decides to cooperate she will have a pay-off of -2.5 m/s or 0 m/s that is going to be preferred more than getting a pay-off of -5 m/s as provoked by an emergency braking necessarily executed for trying to prevent the lane change of the other driver.

### Mobility optimization

We hoped to find optimal behaviors in terms of traffic performance, but as we found out, the best behaviors depend on the density, *i*.*e*. there is no single optimal behavior for all traffic situations. If we analyze traffic performance by observing the flow during the synchronized phase (Fig 11), we notice that the behaviors with the worst results (lowest flow) are those in which the evolution of cooperation was very successful (Fig 8). The model with the best results is the stochastic lane-change behavior model embedded in the GLAI model. Analyzing the results using our mobility measure (Fig 13) we found that our natural behavior model has a better performance. If we focus on its evolution of cooperation results, it has a medium performance. This means that always cooperate or always defect are not efficient driving policies. In other words, we can say that the evolution of cooperation provides adaptation which increases the value of our mobility measure for a given density. This is what we call mobility optimization.

We saw that, during the synchronized phase, there are conditions in which cooperation is a better choice for the agents and other conditions in which agent defection leads to better performance. Paying attention to the lane-changing behavior model section, we can see that defective behavior leads a driver to a greedy space-consumption policy, *i*.*e*., defectors frequently try to cover distances in the shortest time. On the contrary, cooperative behavior leads drivers to a moderate space-generation policy by frequently maintaining velocity or slowing down while facilitating lane changes. By choosing their cooperation probability agents can find a space-generation rate that is sufficient to be exploited by greedy space-consumers, but not enough to form an obstacle for faster vehicles.

Following this reasoning, always defect leads to a fast jam conformation when an obstacle appears on the freeway and always cooperate leads to the generation of big spaces not being utilized.

In cities, traffic rules and norms try to promote or enforce cooperative behavior. However, as we saw, cooperative behavior does not always provide the most efficient mobility. Still, there are reasons for promoting cooperation, such as safety [36] (Fig 17) and psychological health [37].

It is also arguable that a cause of the mobility optimization is the mean velocity`s standard deviation reduction found during the synchronized phase (Figs 9 and 15). However, it seems that this reduction is a consequence of the drivers’ adaptation to their social environment. Finding some insight about why those cooperative rates arise (*i*.*e*. probabilities of the different payoffs for cooperators and defectors) could give us the answer as to what is exactly causing this mobility optimization.

Finally, it is important to mention that all behavior models have the same (poor) flow and mobility performance during the jammed phase, notwithstanding the success of the evolution of cooperation. Because of this, it can be argued that even when driving behaviors are relevant, limiting the number of cars on the streets is even more important for an efficient mobility.

### Future work

The results shown in this paper focused on measures at a global (system) scale. Still, the local (individual) scale is also relevant to complement the ideas discussed above. Obtaining results at a local scale is work in progress and is focused on the value of the payoffs that drivers may get on the games and the probability of obtaining a specific payoff. This could give us enough information to perform a Bayesian game-theoretic formalization of the games and also enlighten us about some of the phenomena reported in this paper: the self-organization occurring during synchronized phases, the conditions that allow the emergence of coherent movement states, and their relation to mobility optimization.

In the context of autonomous vehicles, it would be interesting to define a hybrid balancing strategy between cooperate and defect which would take the best of both extremes (space generation by slower vehicles and space consumption of faster vehicles) to optimize autonomous urban traffic flow.

It is important to mention that although the obtained results are promising we will not go as so far as to label the behavior of the agents of our model as “realistic”. More complex scenarios have been left out of the scope of this paper (like freeways with more than two lanes, freeways with on-ramps and off-ramps) due to limitations of the GLAI model. We believe that it was convenient to start working with the GLAI model even with its limitations rather than to work with another traffic model because many of the internal variables (*D*_*acc*_, *D*_*keep*_, *D*_*dec*_, *lf*, *rf*, *lb* and *rf*) facilitate the integration with the evolution of cooperation framework. The model discussed in this paper may be used as a ground model and improve the scope of the original GLAI model; for example, for tail-gating, more than two lane roads, off-ramps/on-ramps, or crossroads. We will work on widening the scope of the GLAI model, as we hypothesize that drivers will display different behaviors in different segments of the same freeway, *i*.*e*. a driver will behave differently (in evolution of cooperation terms) if she is in a straight segment or if she is in an on-ramp/off-ramp segment.
